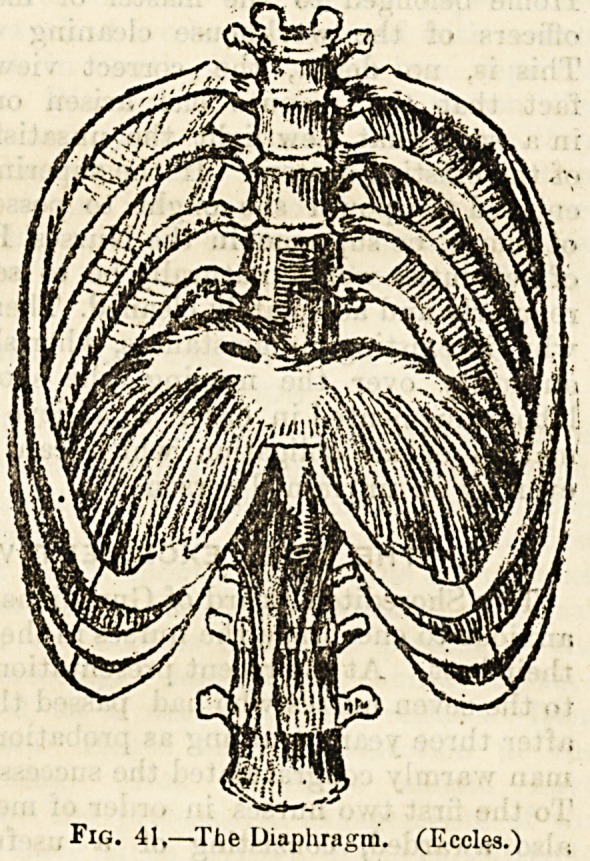# The Hospital. Nursing Section

**Published:** 1902-05-03

**Authors:** 


### The Hospital.

### Nursing Section.

Contributions for this Section of "The Hospital" should be addressed to the Editor, "The Hospital" Nursing Section, 28 & 29 Southampton Street, Strand, London, W.C.

No. 814.—Vol. XXXII. *SATURDAY, MAY 3, 1902.*

### Notes on News from the Nursing World.

### THE ROYAL NATIONAL PENSION FUND FOR NURSES.

A considerable number of policy-holders availed themselves of the cordial invitation of the Council of the Royal National Pension Fund for Nurses to attend the annual meeting. As will be seen from the full report which appears in another part of the paper, the proceedings throughout were as interesting as they were satisfactory. There was nothing for the speakers to explain away, but everything to show that the older the Fund becomes the more it is appreciated, and the wider the area of its operations the more beneficent they are perceived to be. The Chairman, in acknowledging the vote of thanks which was accorded him with much enthusiasm, emphasised a point which cannot be too strongly insisted upon. The Fund, as he said, belongs to the nurses; its success is entirely owing to their co-operation, and though he and all his colleagues manage it gratuitously, the work would be of little use unless the nurses took it up. The steadily increasing support which it receives from them year by year justifies the conviction that they do intend it not merely to maintain its position, but to continue to beat its own record.

### IRELAND'S CONTRIBUTION TO THE WOMEN'S MEMORIAL.

The final meeting of ladies interested in promoting the Women's Memorial to Queen Victoria has just been held at Dublin. A list of the city and county subscriptions was read, from which it appears that the city of Dublin is at the top with £676, and Belfast next with £409; while of counties, Wexford is first with £376, Dublin next with £348, and Down third with £328. The total amount, after deducting all expenses, is £5,855 6s. 4d. In Dublin city Sir John Arnott paid all the expenses, and in Dublin county Lady Meath was equally generous; but elsewhere they are said to have been heavy. No details were, however, supplied. In accordance with the understanding at the outset, the money will not be sent to England, but will be devoted to the augmentation of the Irish branch of the Jubilee Institute for Nurses.

### NORTHERN WORKHOUSE NURSING ASSOCIATION.

As against the unsatisfactory statement in the eleventh annual report of the Northern Workhouse Nursing Association that the committee have been altogether unable to meet the demand for nurses, owing to the serious financial position of the organisation at the commencement of 1901, must be set the fact that the deficit has been reduced from £245 to £28. This has partly been accomplished by the unfortunately essential reduction of the number of probationers in training, but chiefly by the more satisfactory increase of the donations and small subscriptions. It is mentioned in the report that through the generosity of Miss Rathbone the association was able to be represented by a delegate at the Congress of Nurses in Buffalo. The staff now consists of 13 probationers still in training, three nurses on prolonged leave of absence through illness, and of 71 nurses and superintendent nurses holding posts under Boards of Guardians in various parts of the country. The association, at the invitation of the Local Government Board, sent three delegates to give evidence before the Departmental Committee, and it is gratifying to hear that the examination of the delegates was not perfunctory. It lasted two hours, and the committee, it is stated, went most thoroughly into all the points connected with nursing in workhouses in which the association suggested that difficulties had been experienced by its nurses. We hope that the case of the nurse which is quoted in the report was mentioned. This nurse writes: "I am sorry to say I am completely run down. I have been on night duty two and a half months, and have had no proper sleep—some days none at all—my room being in the female imbecile block and next to a children's ward." There is much force in the contention of the association that "it is this general lack of consideration that makes workhouse nursing so unpopular with nurses."

### A MEMORIAL OF SIR HENRY TATE.

The trustees of the Queen's Jubilee Institute for Nurses have received a letter intimating the intention of Amy Lady Tate to hand over to them a capital sum in memory of her husband, the late Sir Henry Tate, sufficient to provide in perpetuity the services of a Queen's Nurse for Silvertown in the borough of West Ham. The memorial is as appropriate as it is acceptable. The late Sir Henry Tate was for many years identified with the densely populated working-class district oddly known as Silvertown, and his name will now be inseparably associated with it.

### A BAD YEAR FOR THE UP-COUNTRY ASSOCIATION.

Misfortunes seem to have dogged the footsteps of the North-Western Provinces and Oudh branch of the Up-country Nursing Association during last year. Three of the nurses left the association, and one after being very seriously ill for six months—so ill that the civil surgeon insisted on having four trained nurses to attend on her—was sent to England, two only remaining on the staff. These two, the report states, "worked splendidly," but with the diminished staff, and house rent and other expenditure continuing much as usual, the daily expenditure was, of course, greater than the receipts. The nursing home was continued through the season at reduced charges, but only one patient came to it, and even current expenses were hardly met by the patients. Naturally, there being no nurses to send to subscribers, the subscriptions have fallen very low. However, it is intended to have a full staff this year, and we hope that the services of the whole of the nurses will be available. Great care should be taken by the committee to engage only those who are physically suitable for work in the trying climate.

### THE SOUTH AFRICAN CO-OPERATIVE SOCIETY.

It will be learnt with interest that the Nurses' Co-operative Society in Johannesburg, whose establishment we recently announced, has already attracted a number of nurses in South Africa who have joined the movement. At an "At Home," held under the auspices of the promoters, the objects of the association were explained to the guests, and it was intimated that an entertainment would be given during Coronation week for the purpose of raising funds.

### A SUCCESSFUL BAZAAR.

The Taunton District Nursing Association has received very substantial assistance from a two days' bazaar which has been held in the Shire Hall on its behalf. At the end of last year there was an excess in expenditure of £48 over income, and the bazaar was organised in the hope of wiping away the deficit, and providing further funds. Owing to the energy with which the enterprise was taken up by every one in the town, the gross receipts of the bazaar amounted to upwards of £250, and the association will thus be placed on a satisfactory financial basis. We hope, however, that there will, in consequence, be no relaxation of efforts to secure regular subscriptions which must always be the basis of enduring solvency.

### TRAINING SCHOOLS IN CUBA.

The general regulations for the schools for nurses to be created in the island of Cuba, with power to issue diplomas to the graduate, comprise several points of interest. The schools may be established in all cities of the island where there are public hospitals containing more than a hundred beds; they are not to comprise less than twenty students; the heads will be the medical director, the superintendent, and the nurses who may be designated to act as assistants to the superintendent; a committee, including a nurse who has previously filled the position of superintendent, will deal with all affairs of a general character affecting the school; the course of instruction in each school will cover a period of three courses of a year each; there will be examinations at the expiration of the first and second courses; the qualifying degrees being awarded by a majority of votes of members of the examination board; there will be a final examination at the end of the third year, after which the diploma to each student whose exercises have been approved will be issued, and she will be admitted to "the practice of the profession of nursing."

### PARIS MUNICIPAL COUNCIL AND THEIR NURSES.

The action of the Municipal Council of Paris in placing before a Special Commission a proposal to fit up on one of the properties belonging to the Assistance Publique a sanatorium of 50 beds for the exclusive use of nurses who may have contracted tubercle in their service, deserves commendation. It might, with advantage, be emulated by our own Local Government Board and by the Metropolitan Asylums Board.

### DEACONESS NURSING HOME, JAMAICA.

At the annual conference of the Deaconess Nursing Home at Kingston, Sister Isabel, in her address, said that the home was founded for the training of women as workers in parochial and hospital work. Afterwards they were to return to their own homes and be provided for by the clergy in charge of their parishes. Funds not having been forthcoming this idea could not be carried out, and the training of parochial helpers had ceased. Several of those trained, she continued, were doing useful work in private nursing, and in hospitals in Jamaica, America, and Cuba. An impression had got abroad that the nursing home was closed because she had resigned her position as head sister owing to failure of her health. This was not the case. The institution was still in vigorous working order, but lack of sufficient money prevented them from training as many workers as they would like. They fully realised that there was much to be done in the streets and lanes of Kingston.

### ARMY NURSES AND MEDAL CLASPS.

A complaint has been made by a South African correspondent of one of the service papers that nursing sisters who have been presented with medals for their work in South Africa have been deprived of the clasps. We understand that the reason why the clasps are not given to nurses is because they are non-combatants. The rule observed in their case applies, we are informed, to civil officials generally, and is by no means confined to nurses. Consequently, there is no question of either a slight or a grievance.

### EXCLUDING DOCTORS FROM A NURSING ASSOCIATION.

The majority of the Auckland Nursing Association have made a mistake in excluding the medical practitioners in the town from the executive committee. It may be conceded that their action in the first instance was not without excuse. The attendances of the members of the committee indicated that last year none of the doctors had been present at the committee meetings, and their non-attendance was construed as a desire to be released from the obligation. Hence their non-election at the present meeting. But allowance should have been made for the professional engagements of the doctors, who have since written to the executive to intimate that if they had wished to be relieved from their duties they would have said so. We observe that in a former communication they declared that if they were to have no voice in the election of, or authority over, the district nurses they should refuse to sanction the services of the latter to any of their patients. This was an injudicious communication, but it does not alter the fact that the medical profession should, if they wish, be represented on the Auckland Nursing Association, and we fear that the refusal of the subscribers to alter their decision will be bad for both patients and nurses.

### HOIST WITH THEIR OWN PETARD.

It is rather amusing that the nurse appointed by the Armagh Board of Guardians under the circumstances mentioned in The Hospital of April 12th, has herself thrown the guardians over after election, and accepted another appointment which, to use her own words, "suited her better." This is the nurse who was avowedly chosen on the ground that she is Protestant, but whose appointment the Irish Local Government Board would not confirm because she could not produce a certificate of proficiency in compliance with the terms of the Nursing Order of July 5th, 1901. Her action shows up the folly of the Armagh Guardians who have now twice chosen nurses who have not taken up the duty. The guardian who, in the light of this fact, suggested that future candidates should be required to produce their certificate of training has, clearly, commonsense on his side.

### KEEPING THE NURSES' HOME CLEAN.

There has been a dispute at Bath as to whether the task of seeing that the Nurses' Home attached to the Workhouse Infirmary is kept clean should devolve upon the matron of the workhouse or the superintendent nurse. The question was discussed at a meeting of the Bath Board of Guardians the other day, and the general opinion was that, according to the order of the Local Government Board, the responsibility of the Nurses' Home belonged to the master or matron as chief officers of the workhouse cleaning establishment. This is, no doubt, the correct view, though the fact that the dispute has arisen only shows up in a somewhat new light the unsatisfactory nature of the existing system. If the superintendent nurse enjoyed the power she ought to possess, she would, of course, be supreme in the Nurses' Home, and one of her duties would naturally be to see that it was regularly and adequately cleaned. There is no reason why, in existing circumstances, when she has not full authority over the nursing, she should have her labour augmented in order to relieve the master or matron of an obligation which can be discharged even by an untrained person.

### THE MORE EXCELLENT WAY.

The Shoreditch Board of Guardians seem laudably anxious to encourage the nurses in their employ to do their best. At the recent presentation of certificates to the seven nurses who had passed the examination after three years' training as probationers, the chairman warmly congratulated the successful candidates. To the first two nurses in order of merit prizes were also awarded, consisting of a useful manual on nursing work and a neat case of instruments. These prizes, apart from their intrinsic worth, will be valued because they afford evidence of a kindly and considerate spirit, which might well be emulated by other Boards.

### DEARTH OF NURSES AT NEWTON ABBOT INFIRMARY.

The hopes of the Newton Abbot Guardians that the difficulty of obtaining nurses would vanish on the appointment of a new superintendent, are not being fulfilled. At the last meeting of the Board the clerk reported that he had "again" advertised for four assistant nurses, but had received no reply. Thereupon one of the guardians proposed that "untrained nurses" should be employed. The chairman, however, said that would be a retrograde step, and it was ultimately determined to advertise again. It may perhaps be the case that Newton Abbot has got rather a bad name among nurses, but this can be lived down in time if the guardians will loyally support the superintendent nurse, and their refusal to entertain a proposal to embarrass her by appointing untrained assistants points to the conclusion that they are willing to do so.

### CORNWALL COUNTY ASSOCIATION.

According to the annual report of the Cornwall County Nursing Association the work has not only been fully maintained but largely increased. Seven Queen's nurses are employed in five districts, and 23 village nurses are engaged in 22 districts. Four nurses are in training, three at Plaistow and one at the Royal Cornwall Infirmary, Truro. Ten village nurses paid 34,881 visits, attended 1,480 patients, sat up 243 nights, and attended 47 operations. The nurses were exceedingly pleased with their visit to the Queen, and on the suggestion of Miss Michie, their superintendent, the Earl of Mount Edgcumbe had written her Majesty to this effect. In reply a kind letter had been received from Miss Knollys saying how pleased the Queen was to hear of their appreciation, and that the nursing of the people was a matter in which she took the greatest interest. The association has received a cheque for £936 17s. 3d. from the Cornish Women's Queen Victoria Memorial Fund, one half of the amount collected in East and West Cornwall, the other half being forwarded to the Central Fund in London. Carnmarth Deanery raised the largest amount, £306, the Powder Deanery, with £290 and Penwill Deanery, with £272, forming a good second and third.

### ANOTHER BOGUS NURSE.

A young woman named Mary Tighe, attired in nurse's uniform, was charged at Birkenhead Police Court last week with having obtained various goods by false pretences. At the shop where she procured the articles she stated that she was well known in the town as Nurse Jones, who lived opposite the Tranmere Workhouse. As the supposed nurse did not pay, inquiries were made, and it was ascertained that the woman was an impostor. We are glad to see that the presiding magistrate commented on the disgraceful nature of the offence "committed under the cloak of a respectable profession," and sentenced the bogus nurse to a month's imprisonment.

### SHORT ITEMS.

The endowment of Holloway Nursing Home forms a portion of the scheme of the Borough of Islington for commemorating the Coronation.—The annual meeting of the subscribers and friends of the East End Mothers' Home, in 394 Commercial Road, will be held on Friday, May 9th. The Bishop of Stepney is to be one of the speakers.—The annual meeting of the members of the Association of Asylum Workers will be held on Thursday, May 22nd, at 4 p.m., at 11 Chandos Street, W., Sir James Crichton-Browne, President, in the chair.—Princess Louise of Schleswig-Holstein paid a visit on Monday to the Royal Free Hospital, Gray's Inn Road, and was shown through the wards and nursing quarters by Miss Wedgwood, the matron.

### Lectures to Nurses on Anatomy.

By W. JOHNSON SMITH, F.R.C.S., Principal Medical Officer, Seamen's Hospital, Greenwich.

### LECTURE XVI.—THE MUSCLES.

THE contractile tissue by which movements of internal organs as well as of the trunk and limbs are effected, is not of the same conformation throughout the human body, but occurs in one or other of two forms, each characterised by differences both in coarse and microscopical appearances, and in the manner in which its function is performed. These two forms of muscular tissue are :—

(1) *Unstriped, or Involuntary Muscular Fibre* found in internal organs, particularly those of a tubular kind such as the intestinal canal, large blood-vessels, and the ducts or discharge pipes of glands. It is arranged in thin layers or scattered bundles of a pale colour. It is called *involuntary* muscle because it acts quite independently of the will. Its ultimate and most minute fibres are made up of rows of long and narrow spindle-shaped cells—the so-called *contractile fibre-cells* (fig. 36).

(2) *Striped, or Voluntary Muscular Fibre.*—This represents the structure of the large and widely-diffused mass of muscle or flesh by which the skeleton is covered. When fresh and living this form of muscle is a red and quivering or contractile substance, which is made up of cords or fibres which, with some little trouble, can be subdivided again and again into smaller strands. The ultimate or most minute fibrous element of voluntary muscle is a narrow band of a ruddy and jelly-like material enclosed in a tube of very thin and transparent membrane, and barred or striped from side to side, whence the name of striped or streaked muscular fibre (fig. 37). This form of muscular structure is called voluntary because its contractile function, as a rule, is under the influence of the will.

To the rule that striped or streaked muscle is under the direct control of the will, whilst the smooth or unstriped muscle acts independently of volition, there are several exceptions, including the important one of the muscular structure of the heart, which consists of striped fibres.

The mass of voluntary muscular tissue, which forms a large proportion of the human frame, is divided into a number of distinct and independent muscles enclosed, and separated from each other by continuous fibrous membranes (*fasciæ*), and by loose cellular tissue and fat. A typical muscle should be made up by striped muscular tissue arranged in bundles for the most part, and by bands or cords of tough fibrous tissue forming *tendons*—"thews" or "sinews." The tendons usually serve to attach the ends or margins of the muscle to bone. The fixed attachment of a muscle is called its *origin*, and the end attached to the bone it moves is called its *insertion*. Each muscle is supplied with blood-vessels and the branches of one or more nerves. Its functional activity, and its utility as a motor organ are dependent upon an uninterrupted communication by means of the supplying nerve trunks, between the muscular structure on the one hand and the brain or spinal cord on the other. The nerve, carrying as it were the mandate of the will, causes the muscular fibres to contract so that the muscle becomes shorter and thicker and its two attached ends are brought nearer together. If the nerve be divided it would fail, like a broken telegraph wire, to carry its

FIG. 36.—Contractile fibre-cells of involuntary muscle. About $\frac{1}{400}$th of an inch in breadth.

FIG. 37.—Striped muscular fibre. Breadth about $\frac{1}{400}$th of an inch.

FIG. 38.—Muscles of back.

FIG. 39.—A, Penniform ; and B, Bipenniform muscles of fingers.

FIG. 40.—Circular muscle of mouth (Orbicularis Oris).

FIG. 41.—The Diaphragm. (Eccles.)

### LECTURES TO NURSES ON ANATOMY.—*Continued.*

message, and the muscle would become paralysed and useless. There is a bewildering variety in the shape of muscles (figs. 38, 39, 40). Some, such as those in front of the abdomen, form thin and very broad sheets; others—these exist in the limbs—are long and narrow masses of muscular tissue, each with a tendon at either end; and between these extremes we meet with many intervening forms due to diversity, not only in the shape and size of the muscle, but also in the arrangement of its bundles of fibres and the relative proportion of its muscular and tendinous constituents. There are square muscles, triangular and fan-shaped muscles, *fusiform* or spindle-shaped muscles, and others in which the fibres are attached obliquely, like the feathers of a quill, along one or both sides of a long tendon—*penniform* and *bipenniform* muscles (see fig. 39).

Some muscles are composed almost wholly of muscular structure and present very little, if any, of the fibrous or tendinous elements. Others, again, present a very small muscular belly and a very long tendon. Several of the elongated muscles in the limbs are divided near their attachments of origin into two, three, or more heads. Hence the terms applied to such muscles of biceps, triceps, and quadriceps. In certain muscles surrounding the entrance to large cavities and canals, such as the mouth, the eye-sockets, and the lower intestine, the muscular fibres are arranged around the orifice in a circular form so as to form a ring (fig. 40).

The function of most of the voluntary muscles, particularly those of the back and limbs, is to maintain the body in the erect position and by acting on the different bones to which they are attached, to perform the various motions which enable us to move from place to place, and to apply to our use and service external objects. In work of this kind the muscle, shortened by its contraction, brings together the different bones to which it is attached. Thus the muscles in front of the neck, as they contract and shorten, bend the head or turn it to one side, and those in front of the forearm bend or flex the hand and fingers.

There are other muscles, however, which act differently and serve other purposes. Some, such as those in front of the abdomen, assist in enclosing large cavities and in supporting the organs they contain. Others, like the *annular* muscles around the mouth and in front of the eyes, enable us to open and close these respective apertures at will. Others, such as those in the tongue, enable us to protrude and withdraw this very movable organ. The soft palate and the upper part of the gullet also contain voluntary muscles which permit us to control to some extent the act of swallowing. The movements of the eyeball are also effected by small but important muscles, the natural and associated action of which may, as in the event of squinting, be disturbed by paralysis or injury of one or other of them.

One of the most important and indispensable muscles in the body is the vaulted partition between the thoracic and abdominal cavities, which is known as the *diaphragm* or midriff (fig. 41). This partition takes a great part in the mechanism of respiration. The outer portions of this extended and *thin* partition are composed of muscular fibres springing from the lower margin of the thoracic cage and from two tendons in front of the spine. These muscular fibres converge to a broad central tendon, the outline of which has been compared to that of a kidney and also to that of a trefoil leaf. As its muscular fibres contract the vault is lowered and the cavity of the chest is deepened from above downwards so as to allow room for the lungs as they are distended by the inspired air. As the fibres relax the diaphragm ascends, and so helps to drive out the air from the lungs in the movement of expiration. These two movements—ascent and descent of the diaphragm—are repeated in the healthy adult from 16 to 20 times per minute. As the diaphragm descends during the taking in, or inspiration of air, the front wall of the abdomen is pushed forwards. In the diaphragm there are three openings: one allowing the inferior vena cava to reach the right auricle of the heart, the second for the passage of the gullet or œsophagus just before it reaches the stomach, and the third, which is called the aortic opening, marks the boundary of the thoracic and abdominal portions of this arterial trunk. This muscle, which, with regard to its functions may be regarded as an involuntary one, and beyond the control of the will, takes part in the expression of the emotions, in laughing, crying, and sobbing, and is moreover the chief factor in the occasional troubles of vomiting, coughing, and hiccup. As Sir Charles Bell pointed out, "in sickness and oppression, lowness and sighing, in weeping and laughing, in joy or in fear, all our feelings seem to concentrate in this part."

### Appointments.

[No charge is made for announcements under this head, and we are always glad to receive, and publish, appointments. But it is essential that in all cases the school of training should be given.]

Blackpool Infectious Diseases Hospital.—Miss A. Meek has been appointed night charge nurse. She was trained, for three years, at Leeds General Infirmary, and was previously for two years at Hull Sanatorium.

Coventry and Warwickshire Hospital.—Mrs. Florence Lucas has been appointed lady superintendent and matron. She was trained at St. Thomas's Hospital, London, where she was afterwards sister of the adult and children's wards, and of the operating theatre. She was subsequently matron and superintendent of the Royal Devon and Exeter Hospital, Exeter.

General Hospital, Weston-super-Mare.—Miss Edith Hobbs has been appointed charge nurse. She was trained at Teignmouth Hospital and at the Royal United Hospital, Bath. She has since been on the private nursing staff attached to the latter institution.

Hackney Union Infirmary.—Miss Emilie Louise Foskett has been appointed assistant matron. She was trained at the East Sussex Hospital.

Lewisham Infirmary.—Miss Beatrice Ellen Denyer has been appointed sister. She was trained at Lincoln County Hospital, and Guy's Hospital, London. She has since been nurse in the Government Hospital, Umtali, and charge nurse at the New Somerset Hospital, Capetown.

Louth Hospital.—Miss Maude Crichton has been appointed matron. She was trained at the Leeds General Infirmary, and has since been night superintendent at the Royal Infirmary, Hull.

Monkwearmouth and Southwick Hospital.—Miss Fannie M. Smithies has been appointed matron. She was trained at Bolton Infirmary, where she was afterwards staff nurse for six months. She has since done three years' private nursing in connection with Fitzroy House, London, has been charge nurse at Huddersfield Infirmary (children's and male medical wards and operating theatre), four years; sister, male, surgical, and accident wards, at Cardiff Infirmary, eight months; matron of Bridgend Cottage Hospital, thirteen months; assistant matron at the Royal Infirmary, Hull; and a year matron of Altrincham General Hospital.

Refugee Camp Hospital, Winburg, Orange River Colony.—Miss Florence Hird has been appointed matron. She was trained for three years at Montrose Royal Infirmary, and afterwards did private nursing at Shrewsbury Nursing Institute. Since then she has done district work, and has had charge of a surgical home in London.

The Hospital Village Home for Girls, Ilford.—Miss F. A. Harding has been appointed sister-in-charge. She was trained at the Adelaide Hospital, Dublin, where she afterwards held the post of out-patient sister and masseuse for three years, also of assistant matron. She is a member of the Army Nursing Reserve, and was for nearly a year and a half nursing sister at the Station Hospital, Devonport.

### Royal National Pension Fund for Nurses.

THE GREATEST PROVIDENT ORGANISATION FOR WOMEN IN THE WORLD.

THE fifteenth annual general meeting of the members of the Royal National Pension Fund for Nurses was held at River Plate House, Finsbury Circus, on Thursday, April 24th, 1902, under the presidency of Everard A. Hambro, Esq. Supporting the Chairman were the following Members of the Council:—Sir Henry Burdett, K.C.B.; Mr. R. Ernest Alexander; Mr. Thomas Bryant, F.R.C.S.; Mr. Walter S. M. Burns; Mr. Thomas Charles Dewey, F.I.A.; Dr. S. H. Habershon; Mr. C. Eric Hambro, M.P.; the Hon. Egremont J. Mills, D.S.O.; Mr. J. Pierpont Morgan, jun.; Mr. Edward Rawlings; the Hon. Walter Rothschild, M.P.; Mr. Charles W. Trotter, and Mr. Falconer L. Wallace. Amongst those present were the Hon. Herbert C. Gibbs; Mr. George King, F.I.A., F.F.A.; Mr. Perceval A. Nairne (Chairman Seamen's Hospital, Greenwich); Miss Mabel Cave (Matron of Westminster Hospital); Miss L. M. Gordon (matron of St. Thomas's Hospital); Miss K. H. Monk (matron King's College Hospital); Miss Vincent (late matron of St. Marylebone Infirmary); Miss Pritchard (hon. secretary of the Junius S. Morgan Benevolent Fund); Mrs. Farmer (secretary of the Junius S. Morgan Benevolent Fund); Miss H. A. C. Gordon (matron of Charing Cross Hospital); Inspector-General Woods, R.N., M.V.O., etc., and many policy-holders.

The CHAIRMAN, in opening the proceedings, said: The secretary will kindly read the notice calling the meeting.

The SECRETARY (Mr. Louis H. M. Dick), having read the notice, then announced, by request of the Chairman, that letters of regret for non-attendance had been received from Dr. H. P. Hawkins, Dr. G. W. Potter, Mr. Alfred J. Waley, and Miss Armit.

The CHAIRMAN: It is now my pleasure to move "That the report, accounts, and balance-sheet be received and adopted." This is the first time I have had the pleasure of meeting you here, as on previous occasions I have been on the Continent when you have met, and Sir Henry Burdett has generally taken the chair. I am afraid that you will find me a poor substitute for him, as I am quite unable to address you as he would do; but the report is so full that everything really is clearly laid before you in it, and very little is left for me to say. The little that is left for me is really only to convey my thanks to those who continuously assist us on the council in the management and the furtherance of the objects of the Fund. I should like, although it has been already done, to thank the staff for their great energy in their arduous duties; and I should like again to thank the secretary for devoting, as he does, the whole of his energy and life to the furtherance of the Fund.

SOUND FINANCIAL ADVICE ALWAYS OBTAINABLE.

I should also like to thank all those gentlemen who have always assisted us on every occasion when we have asked for their advice in the management of our investments—gentlemen whose names do not appear in the report. Whenever I have wanted any assistance from any financial gentlemen in the City respecting a security we contemplated investing in, I have found that the best and fullest information has been given me on the subject, and I have further found that if we decided to buy anything, every broker in the City was willing to work for us for nothing. In fact, wherever I go, I find a desire to assist us in every possible way. When houses issue loans of which we desire any portion, I always find, however large the premium is, that as soon as I mention it is for the Nurses' Fund, we are immediately allotted in full whatever we ask for, and sometimes we receive back the commission. In fact, my duty as chairman of this fund has always been more to thank others for assisting me than to do anything myself. There is one other body which I should like to thank—namely, our critics. Like every fund that has prospered, we sometimes have critics, and however careful we may be ourselves to do everything that we think right, there is no doubt that criticism is a very good thing. We are always careful, but when we are criticised we perhaps look more carefully into our arrangements than we might do if we were not criticised.

SUBSTANTIAL INCREASE IN VALUE OF SECURITIES.

I am thankful to say that on the occasion when we were last criticised, I looked into everything I could; and, although, owing to the unfortunate war, securities generally have gone down, yet after inquiring about the investments we had made for the Nurses' Fund, I found that although some of them had fallen in price, yet if they had all been realised at the official quotation, a substantial benefit would have been shown to the fund on their cost. We value those who criticise us, and we look into their criticisms carefully, but we have found that our action —although we must make mistakes—has been so far prosperous on the whole. I really, ladies and gentlemen, have nothing to add. I can but again refer you to the report, which gives you the fullest information, and I should like again to thank everyone who has assisted us in our work. It is a work of love, which everyone is willing to assist us in, and it is a work which I hope we may be able to carry on successfully to the end. (Cheers.) I now formally move "That the report, accounts, and balance sheet be received and adopted."

Sir HENRY BURDETT, K.C.B.: I have much pleasure in seconding the resolution. I can fully endorse all that has fallen from the chair with respect to the value of the services which are rendered by the City of London to this Fund. In fact, if it had not been for the support with which this Fund was from the first received, and which has been given to it by the City from its foundation to the present time, it would have been impossible for it to have attained the proud position it is in to-day—that of being the greatest insurance fund for working women in the world. It is to me—as it must also be to the Chairman—a great satisfaction to realise that we have on the platform to-day representatives of everybody who gave the original £20,000, and, in the person of Mr. Hambro, one of the four generous donors. (Cheers.) That is a great fact, and the pleasure is strengthened by the knowledge that the representatives of the other founders are in most cases their eldest sons. (Cheers.). All of them are here to show their personal interest, and the interest of their firms in the fund, and as members of the council most of them give their personal service to it and valuable assistance too in many ways. Surely, from the nurses' point of view, from the point of view of the managers of institutions in this country who train and employ nurses, they could not have a better guarantee for anyone who joins a fund of this kind, or a stronger ground of confidence in it, than that the Pension Fund is supported by the continued personal services of the leading financiers in the City of London. Nurses and officials may be grateful for the opportunity of being able to join it. (Hear, hear.)

WORK OF BENEVOLENT FUND.

I have always, in seconding the report, dealt not with the figures, which are left to the Chairman, but with the Benevolent Fund. There can be no doubt that if Mr. Junius S. Morgan knew—possibly he does know—how his memory in connection with this fund has been commemorated by the establishment of this Benevolent Fund, and he judged the memorial by the work it does, he would be perfectly satisfied. We have on the Benevolent Fund at the present time of the older nurses nearly 50 who are

### ROYAL NATIONAL PENSION FUND FOR NURSES.—*Continued.*

permanently pensioned. Lady Rothschild is president of the committee of the Benevolent Fund, and her personal interest in it is shown by the fact that she has never, from its institution to the present time, failed to be present and to preside at the meetings of that committee. (Cheers.) There is in connection with the Benevolent Fund this very important fact—that not one of the policy holders in the fund can permanently fall out by the way, or be stricken down, who is not taken in hand by the Benevolent Fund and effectively provided for. I do not think it is necessary, or even desirable, that I should detain you by making a long speech to-day; but I do feel, as we have been thanking the gentlemen in the City, and the members of the council who represent the large firms, for their services to the general fund, that we owe a great debt of thanks to Lady Rothschild and the ladies who are associated with her in the management of the Benevolent Fund. (Hear, hear.) Whatever claims the general Fund may have for the efficiency of its administration, I feel that the way in which the ladies have administered the Benevolent Fund entitles them to similar recognition for equal efficiency and equal success in their work. (Hear, hear.)

The CHAIRMAN put the resolution to the meeting in the usual way, and it was carried unanimously.

The Hon. Herbert C. Gibbs and Mr. George King were then appointed scrutineers to examine the votes received for the election of representatives of the annuitants and policy holders of the society as members of council.

The CHAIRMAN: The retiring members of the council this year are Sir Henry Burdett, Mr. Edward Rawlings, Mr. A. H. A. Morton, M.P., Mr. Eric Hambro, M.P., and Mr. Walter S. M. Burns. These gentlemen being eligible for re-election, I will ask Mr. Bryant if he would kindly propose them and if Mr. Mills will second them.

Mr. THOMAS BRYANT, F.R.C.S.: It is with much pleasure that I rise to propose the re-election of the gentlemen whose names have just been mentioned. Sir Henry Burdett was the original founder of the Fund, and all these gentlemen have been good workers from the very beginning and representatives of the founders, as you have heard Sir Henry Burdett say. For my own part, I do not think any of us who have the privilege of doing any work for this Fund can speak too highly of those who have conducted it. Its birth was very difficult and its future was very uncertain, but still the most sanguine hopes of those who started it have been realised. (Hear, hear.) I was not one of the most sanguine when it started, and I can only say that I am astonished at the great success with which the Fund has been attended. I congratulate all the members of it, and I can only ask each nurse who has joined it to use her best influence to persuade her sister nurse to come into the Fund. There is nothing like it in the country; there is nothing like it that can so help women and produce more hopeful feelings in their work than to realise that they are members of such a pension fund as this. Indeed, you can use no words too strong to influence those around you to join you in it. As to the work which these gentlemen have done, I will say nothing. You know by the success of the institution that the work has been sound from the beginning. I have great pleasure in proposing the re-election of the retiring members of the council—Sir Henry Burdett, K.C.B., Mr. E. Rawlings, Mr. A. H. A. Morton, M.P., Mr. C. Eric Hambro, M.P., and Mr. W. S. M. Burns.

The Hon. E. J. MILLS, D.S.O.: I have much pleasure in seconding the motion.

The resolution was carried unanimously.

Mr. THOMAS C. DEWEY, F.I.A.: Mr. Chairman, I beg to propose that Messrs. Whinney, Smith, and Whinney be elected auditors for the ensuing year, and that the best thanks of the meeting be given to Mr. Whinney for his services in the past year. I am sure we cannot forget that we owe a deep debt of gratitude to Mr. Whinney for acting as our auditor for so long for such a nominal consideration. The figures continue to grow, and not only are the figures large, but the amount of detail on both sides of the revenue account is very heavy and has taken an immense amount of time to examine. I beg to move that the best thanks of this meeting be given to Mr. Whinney for his services in the past year, and I also propose the appointment of Messrs. Whinney, Smith, and Whinney as auditors for the ensuing year.

Mr. R. ERNEST ALEXANDER: I have much pleasure in seconding that.

The CHAIRMAN: It has been proposed and seconded that Messrs. Whinney, Smith, and Whinney be elected auditors for the ensuing year, and that their remuneration be fixed at 50 guineas. I should like myself to say how much we owe to those who have taken upon themselves the most arduous task of auditing the accounts. He then put the resolution to the meeting, and it was carried unanimously.

### ELECTION OF POLICY-HOLDERS' REPRESENTATIVES.

Mr. KING then announced the result of the scrutiny for the election of representatives of the annuitants and policy-holders as members of the council. He stated: Of the cards issued 1,301 were received, and 30 were spoilt and contained more than seven names. For the seven names issued there was a very large preponderance of votes, namely:—Miss Mabel Cave, 1,229; Miss E. Fisher, 1,226; Miss L. M. Gordon, 1,247; Miss K. H. Monk, 1,226; Miss E. M. Shirley, 1,200; Miss S. A. Swift, 1,233; Miss E. Vincent, 1,202. Miss Peter, who follows next, has 12 votes, three ladies 4 each, and 19 ladies received fewer votes. Unfortunately, Miss Shirley has died since the cards were issued, so her name necessarily drops out, and presumably the other six candidates in the printed list and Miss Peter are elected.

The CHAIRMAN declared Miss Cave, Miss Fisher, Miss Gordon, Miss Monk, Miss Swift, Miss Vincent, and Miss Pauline Peter duly elected, and directed that the ballot cards be destroyed.

### VOTE OF THANKS TO CHAIRMAN.

The Hon. W. ROTHSCHILD, M.P.: Ladies and gentlemen,—A most pleasant task has fallen to my lot, and I have the very enjoyable duty of asking you to pass a most cordial vote of thanks to the Chairman, both for presiding here to-day and also for all that he has done for the Fund in the course of the past year. (Hear, hear.) I am sure you all know the immense interest he takes in the Fund and in everything connected with it; and although in his speech he said that he had only to thank others for doing the work and that he had done nothing himself, I am sure you will all know that that is not literally the fact, and that wherever it is possible for him to do work connected with the Fund, he does it most efficiently. I ask you, therefore, to pass a most hearty vote of thanks to the Chairman, and I express the hope—which I am sure you will echo—that he will remain our chairman for a great number of years. (Hear, hear.)

The Hon. HERBERT C. GIBBS: Perhaps, as a former member of the council, I may be considered an appropriate person, in some way, to second this vote of thanks. I am rather ashamed to say it, but I certainly showed more appreciation of the work and the responsibility of the council than anyone else, because I resigned the position I held, being unable to find the time to attend to the duties properly. When I remember your able and energetic predecessor in the chair, under whom I had the honour of

### ROYAL NATIONAL PENSION FUND FOR NURSES.—*Continued.*

serving, I must say that I know perfectly well that what Mr. Rothschild said is correct—and I thoroughly agree with him—that the work of the chairman is very arduous and responsible; and there is certainly no one who would deny that our present Chairman is as well fitted for it as anyone in the City of London. (Cheers.) I have much pleasure in seconding the vote.

Sir Henry Burdett: In putting the resolution, I should like to add my tribute to the really invaluable service which Mr. Hambro has rendered to this Fund. (Hear, hear.) He took the chair most kindly and most self-denyingly when we were left in very great difficulty by the lamented death of my dear friend, Mr. Walter Burns, who from the outset had devoted his energy and time to promoting its success. It was of the first importance that someone of great authority and great influence, who was universally esteemed in the City of London, should succeed Mr. Burns as Chairman of this Fund. Mr. Hambro came forward when he was invited—(cheers)—and accepted the position—an act, I know, of infinite self-denial. I need not tell you, and I need not tell the City, that in the discharge of the duties attaching to the office—difficult as they were and always are—we have found in Mr. Hambro a chairman for whose services it would be difficult, if not impossible, really to exaggerate our indebtedness. I am quite sure that I am only echoing the feeling of every member of the council—(hear, hear)—in saying this. Mr. Hambro is so very modest, that while it is a great pleasure to testify to his services, I feel that it is also a duty to do so. I now put the resolution that the most cordial vote of thanks of the nurses and the members of the council be given to Mr. Hambro.

The resolution was carried by acclamation.

The Chairman: Mr. Walter Rothschild, Mr. Gibbs, ladies, friends, and gentlemen, I venture to put in the word "friends" because in my life I have received so much friendship from nurses that I hardly ever pass one in the street without feeling that I pass a friend. That feeling was what first influenced me in associating myself with the Fund. I want the nurses, however, to remember that however energetically we may work, and however successfully this Fund may have been started from our friendship towards the nurses, it is now a Fund that belongs to them themselves. By their co-operation they have increased the Fund, which started with only £20,000, to a very large sum indeed. That is entirely owing to their co-operation. It is perfectly true that we manage it, working *con amore* and for nothing. We work it for our love of the work, but our work would be of little use unless the nurses took it up.

Fund not a Charity.

Remember, it is not a charity in any sense of the word, it is not a pension fund in any sense of the word—it is a business annuity, which you can all carry to very large success if you are willing to do so. It is really a business—an annuity created originally, I grant you, by friendliness to the nurses, but which they have now themselves built up to a point where it stands on its own legs, and can go forward with very great success and with very great assistance to the nurses themselves if they will help it. I thank you very much for the kind way in which you have received the proposal of Mr. Rothschild and Mr. Gibbs, but I beg of you to remember that whatever we do—and in my first speech I thanked those who have helped the Fund for their services—that whatever we do, it is the nurses we must thank for the success of the Fund (Cheers.)

The proceedings then terminated.

### The Royal British Nurses' Association.

### THE MIDWIVES BILL.

It is some time, owing to various causes, since a quarterly council meeting of the Royal British Nurses' Association has been honoured by the presence of its Royal President, Princess Christian was, however, able to be in the chair on Friday afternoon, and there was a full attendance of council members to meet her. The Princess was attended by Mrs. Coster, the nurse honorary secretary. With her at the table were also Mr. Pickering Pick, vice-president; Mr. John Langton, hon. treasurer; Mr. Fardon, hon. secretary; and Miss G. A. Leigh, secretary.

The Princess having signed the minutes of the previous meeting, Mr. Langton moved the adoption of the financial report for the quarter, showing that receipts amounted to £401 0s. 10d., expenditure to £438 7s. 11d., and that there was a bank balance of £50 16s. The total amount received towards the Settlement Fund was £2,126 17s. 10d., including £1,103 4s. 5d., proceeds of the sale held early in the year at 24 Park Lane; £2,000 of this sum had been invested in trust stocks. Mr. Langton reiterated his opinion that no beginning must be made until the funds justified expenditure on building, for although, as he explained, the resident nurses would have to find most of their food, clothing, etc., the home would have to be kept up. He thought that £100 a year at least should be in hand before a start was made. That amount would keep it going, and would include a caretaker. He would discourage a beginning being made for some time yet. Mr. Pickering Pick seconded the adoption of the report, and it was agreed to.

Mr. Fardon next read the report of the Executive Committee. Twenty-two nurses had been registered, 21 had been elected members, five had withdrawn, and three had died. Besides the sale already alluded to in the financial report one was held, by the kindness of Mrs. Tawney, at Oxford when £17 15s. was realised for the Settlement Fund. Mrs. Harbin, matron of the Children's Hospital, Glasgow, and Miss Georgina Hopper, matron of the Somerset Hospital, Capetown, had been appointed to represent the association in their respective localities. An allusion to the Midwives Bill and to the date fixed for the annual meeting (June 16th at noon) closed the report.

The Midwives Bill.

The greater part of Mr. Fardon's speech in submitting the report dealt with the paragraph at the end, which was as follows: "The Midwives Bill has been read a second time and considered in Committee. It is set down for the third reading early in June, when efforts will be made to amend it. In the event of these being unsuccessful and the Bill passing in its present form, the arrangements for the efficient training of the midwives it proposes to register are so inadequate that this association could not be identified with the measure." Mr. Fardon said that he had pointed out to one of the Members of Parliament whose names appeared on the back of the Bill, that it contained no provision for the training of midwives whom it was proposed to place on the register, and that the Association, and indeed everyone acquainted with the high value of adequate training in the care of the sick, thought that some such provision should be made. The member alluded to thought this was a matter that might be left to the Central Midwives' Board, which would, no doubt, see that everything essential was done. He sympathised with the view that some representative of the nurses should sit on the board. The Bill

### THE ROYAL BRITISH NURSING ASSOCIATION.—*Continued.*

passed its second reading, and was referred to the standing committee on law. It was then stated that the Association had opposed the Bill. This was not true.

Amendments Required.

Certain amendments were proposed to improve it, and that was all. A letter was received, after the second reading had been passed, which read as follows:—"I have persuaded our friends to insert a clause and put a representative of the Royal British Nurses' Association on the Central Midwives' Board." This showed that the Association did not oppose the Bill. Mr. Fardon said he was quite aware that there was a feeling that this was a matter which in no way concerned the Association. He was afraid, however, that the Bill had been drawn up and promoted by those who had no practical acquaintance with the work of a midwife, and he addressed himself to the nurses present who understood the principles of aseptic nursing. They knew the danger that lurked in dirty hands and dirty dresses, and they knew that it was not the want of skill in treatment so much as the risk of infection to mother and child that was the important matter. The Association had endeavoured to establish some form of guarantee of efficient training, and now they were told that training was a matter to be left with the Central Midwives' Board, the constitution of which he proceeded to explain. The Board would have no option as to placing on the register all who presented the necessary certificates. One of these might be granted by the Royal College of Physicians of Ireland; as, however, the Bill did not apply to Ireland, and as, moreover, that body had for some time ceased to grant certificates to midwives, the intention was not quite clear.

The L.O.S. Certifcate.

What was of more importance was that the Board was also bound to place upon its register every applicant holding the certificate of the London Obstetrical Society, and in addition, everyone who might obtain that certificate within the first two years after the passing of the Act. The London Obstetrical Society thus held the key of the register for two years, and the point was this, that it was a body which did not train midwives, it only examined them. Some of the candidates, like those whom he was addressing, took their L.O.S. after three years' general hospital training; others took it after attending twenty cases and witnessing twenty more. Were these trained women to be placed on a level with those who had never done any work in a hospital or a well-managed workhouse infirmary? Could training given in cottages qualify any woman as a midwife? "I ask you," said the speaker, "how much you can pick up in twenty cases? Yet the Board is to be compelled to admit these persons, whether they like it or not." He had been told that 7,000 women held this certificate, and that it had been granted for twenty years. There must be some, if only one or two per cent., of incompetents in all that number, and they ought not to be placed upon any register whatever. Yet the Board would have no choice. How would it affect the nurses of this Association, and others who were highly qualified? All the members of the R. B. N. A. had spent three years in a well-appointed hospital, and had, in addition, taken out their certificates in monthly nursing. Was it likely that such persons would wish to place themselves on an equality with those who had had no more training than that he had described? It might be said that they need not be on the register if they did not wish to be.

Practising without Registration.

Well, they might certainly practise without registration, but by putting anything on their professional cards to show that they were qualified to practise, they would render themselves liable to a £5 fine. But it appeared to him that the very roll issued by the Association rendered them liable to that penalty, for it was stated there, opposite every nurse's name, what certificates she had taken. Many wished to practise monthly nursing only, and not midwifery, but they wished it to be known that they were qualified to do both. They were assured that the idea was to protect poor women from dirty and careless treatment; he very much doubted whether the Bill would be an efficient remedy. He was told that a great many women with the slight training to which he had alluded were undertaking monthly nursing, and calling themselves "Ladies' Nurses." As a body of nurses the Association had done more than any other body to publish the importance of high training, and he thought it had had an effect all over the world. The hospitals abroad and in the colonies were beginning to give a three years' training, and a Bill which set forth such a wretchedly low standard did not deserve support. A member of Parliament had said that these women were not nurses, but it was surely as nurses that they would wish to pose. "As an Association," the speaker concluded, "we felt that we could not give our sanction to a Bill which did not provide in some way for the training of midwives, because we considered that if it did not do this, instead of being a blessing it would be a source of danger. We found we were unable to amend the Bill, or to get those who were responsible in any way to listen to suggestions, and as we could not give any assistance to the Bill, we withdrew."

The Discussion.

Dr. Galton opened the discussion which followed. He confined his remarks to the concluding paragraph of the report on general grounds, and moved that the words, "In the event of these being unsuccessful, and the Bill passing in its present form, the arrangements for the efficient training of the midwives it proposes to register are so inadequate that this Association could not be identified with the measure," be omitted. If the Association preferred an amended Bill, he said, let it try to get it amended. No one in that room, he imagined, had any doubt that the Bill would pass. He hoped that no such futile thing as the concluding paragraph to the report, which was of no earthly use to the Association or anyone else, would go out to the world. It was like children at play saying, "If we can't have it all, we won't have anything to do with it." He held that it would be very wrong to send it out. The Association had lived a quiet life lately, and this would plunge it into controversy. "The Bill," said Dr. Galton, "has nothing to do with our women and I move that the paragraph be omitted."

The motion was cordially seconded by Mr. Latter.

Dr. Godson agreed that if the Bill became law it would be wrong not to submit to it. A Bill, though faulty, was better than no Bill at all. It gave opportunities for the future. He felt that there were many difficulties, but he hoped there was time to remedy them.

The next speaker was Dr. Thorne. He said he was to a great extent in harmony with the two previous speakers. The Association occupied a very proud position with regard to the question. There was nobody in the Kingdom to be compared with the midwives of this Association. Those women with short training knew nothing of aseptic nursing. But if the Bill became law, it would have to be respected throughout the country, and it was an important question whether the Association should ignore it, or endeavour to raise the midwives of the country to their own standard. His own opinion was that they might do good by claiming a

### THE ROYAL BRITISH NURSING ASSOCIATION.—*Continued.*

seat on the Central Midwives' Board, and taking their stand as advocates of higher training.

Princess Christian then put the amendment, five voted for it and four against. "The paragraph, then," she said, "will be omitted. Is Dr. Godson of opinion that we might get a seat on the Board?" Dr. Godson replied that he was.

The next speaker was Dr. Barker. He proposed to substitute the phrase, "That in the event of this Bill becoming law, this Association pledges itself to make every effort to have the law altered." There was no disloyalty, he said, in wishing to have a measure altered. Dr. Thorne followed. He thought that something should be done at once, and he suggested that a deputation should go from the Council, asking for representation on the Central Midwives' Board as a right. This was seconded by Mrs. Dacre Craven, and Dr. Thorne then submitted his proposal in the form of a resolution, "That a deputation be appointed by the Council of the Royal British Nurses' Association to wait upon the Lord President of the Council, and to claim for the Association a seat on the Central Midwives' Board." This was carried, and the quarterly report, minus the disputed paragraph, was adopted.

### A Distinctive Cape.

The next business on the agenda was to consider a motion by Miss James: "That it is desirable that a distinctive cape be designed and issued by the Royal British Nurses' Association to members desirous of wearing it."

Miss James, who was wearing her uniform, stated her reasons for bringing forward the motion; the subject, she understood, was not a new one, and she believed the proposal had more than once been discussed. She and others felt that, owing to the present abuse of the uniform, some distinction was desirable. Much as they appreciated the badge they wanted something more. A cape to be procured only from one firm, and on an order from the secretary, she felt might meet the case. If the wearing of such a cape interfered with a hospital uniform, she would suggest an embroidered crown, to be worn over the letters R.B.N.A., in a conspicuous place on the cape. The Dublin nurses wore one, and everyone knew who they were.

Mrs. Dacre Craven said a special dress was certainly wanted, and she gave an instance of the abuse of uniform which had come under her own observation—it had been reported that a "nurse" was frequenting the music-halls. She had taken legal advice on the matter, and was told that unless it could be proved that the woman made money by her use of the dress nothing could be done. She only wished it could.

The Princess suggested that a small committee should be appointed to discuss the matter and to report to the council, and the following were nominated:—Mr. Pickering Pick, Mrs. Dacre Craven, Mrs. Coster, Mrs. Latter, Miss Thorold, and Miss James.

### The Metropolitan Nursing Association.

### ANNUAL MEETING AT GROSVENOR HOUSE.

There was an additional attraction this year at the annual meeting, at Grosvenor House, of the Metropolitan Nursing Association, for the committee were fortunate enough to have their new President, Her Royal Highness, Princess Christian, in the chair. The meeting, which was in the large ball-room, took place on Thursday afternoon, and there was a very good attendance; for although, at the moment of the Princess's arrival, there were several rows of empty chairs, they filled up before many minutes had passed. The Rev. A. B. Peile, Master of St. Katharine's, the Rev. Dacre Craven, Hon. Secretary of the Association, Mr. Henry Bonham Carter, Vice-Chairman, and Mr. F. D. Mocatta supported Her Royal Highness on the platform.

Mr. H. Bonham Carter elicited very hearty applause by the announcement that H.R.H. had graciously consented to accept the position of President; it was the first time the Association had been so honoured by a member of the Royal Family. He next moved the adoption of the twenty-sixth annual report, opening with an allusion to the great loss sustained by the Association in the death of Mr. William Rathbone, who, as well as being among the first to give practical effect to Miss Nightingale's views, was, until the day of his death, a member of their executive committee.

A sketch of Mr. Rathbone's work in connection with the Nightingale School for Nurses at St. Thomas's Hospital, the reforms at the Royal Infirmary at Liverpool, and the scheme for providing district nurses in that city, followed, and in 1874, on the initiative of the Order of St. John of Jerusalem, the Metropolitan and National Nursing Association for providing trained nurses for the sick poor in their own homes was founded. Largely at his own expense, Mr. Rathbone instituted an exhaustive inquiry into the actual state of London with regard to district nursing, and in the scheme recommended, prominence was given to the necessity, as an adjunct to hospital training, for special training in district nursing, the supervision by superintendents, the establishment of homes, the restriction of the nurses' work to patients attended by a medical man, the enforcement of her strict obedience to his orders, and lastly the selection of district nurses from a better educated class of women.

This led Mr. Bonham Carter up to a graceful allusion to the work of Miss Florence Lees (Mrs. Dacre Craven) as superintendent-general when the first home was opened in 1875; it was at her suggestion, and through the influence of the late Duke of Westminster, that the decision was made that the nurses of the association should be taken from the better educated classes.

### The Year's Work: the Nurses.

During the year 1901, 15 district nurse probationers were admitted, and there were ten already on the books; of these, 17 completed six months' district training, and eight remained on the books at the close of the year. All had received two years' general training before admission, and on the completion of their training they received appointments under the Q.V.J.I. in the service of its affiliated associations. The demands for army nurses and the inducements offered by private nursing made it very difficult at times to find suitable candidates. The usual courses of lectures, provided by the Queen's Institute, had been given, and examinations were held after each course. The subjects were: "Obstetrics and Diseases of Women," by Miss Appell, M.D.; and "Hygiene," by Mrs. Goslett, member of the Sanitary Institute. A course on "Sick Cookery" was also given by the staff teacher at the National School of Cookery. By arrangement with the authorities of St. Bartholomew's Hospital, the nurses attended the lying-in cases visited by the maternity students of the hospital, and by this means gained most valuable experience, which could not be obtained in any other way.

### The Patients.

The number of patients attended was 1,285, of which 1,192 were new cases. The total number was an increase of 159 on the previous year. Of these 828 recovered or became convalescent, 135 were transferred to a hospital or infirmary,

### THE METROPOLITAN NURSING ASSOCIATION.—*Continued.*

207 died, 16 were removed from the books, and 99 remained on December 31st, 1901. The majority of the cases nursed were women, next came children (of whom there were 248, not including the maternity cases from St. Bartholomew's Hospital), and 167 were men. At the request of the medical staff a nurse attended daily for one hour at the Bloomsbury Dispensary, and the Farringdon Dispensary, Holborn, as well as twice a week at the Finsbury Dispensary. Subsequent visits to these patients at their own homes, by the request of the medical men, were of the very greatest service to the sufferers. The bulk of the cases were such as could be as well nursed, and frequently better nursed, in the patients' own homes than in hospital or parish infirmary, and a large number, probably the majority, would not be admitted to a hospital. Patients were attended in 64 ecclesiastical parishes within a radius of a mile and a half from the home.

### Finance.

Referring to financial affairs, Mr. Bonham Carter said that the total expenditure amounted for the year to £1,769 10s. 10d. It exceeded the previous year by £333, and this excess called for some explanation. Under the heading rent, rates, taxes, and insurance, there was an increase of £147 1s. 4d., arising from the increased value of the building; there was also the interest on a loan from the bankers to meet the cost of the additions to the buildings, £30 16s. 8d. (this would diminish as the loan was gradually paid off); an item for uniforms, £58 10s. 3d., which should have been included in the previous year's account; while £24 3s. 7d. was accounted for in the increased expenditure arising from the additions to the house in Bloomsbury Square, in stationery, advertisements, etc. This gave a total of £340 7s. 5d., but by certain small economies it had been reduced to £333. On the receipt side, donations, subscriptions, and payments from the Q.V.J.I. (including balance brought forward and excluding loan of £200 repaid from the building account) amounted to £1,398 15s. 8d., being £274 less than in the previous year. The excess of expenditure over receipts (£370 15s. 2d.) had been met by absorbing £200 (the loan repaid), and borrowing £200 from the bank, leaving a balance in hand of £29 4s. 10d. The building account was closed at the end of the year with the exception of one item, which had since been paid out of the current account, and there remained the loan of £1,300 due to the bank. The original estimates had been exceeded by a little over £100, on the whole a satisfactory result. The thanks of the committee were due to the superintendent and staff nurses for the indefatigable way in which the Home had been carried on, and he would earnestly ask for the increased support of an institution which was of such incalculable benefit to the sick poor.

The motion having been seconded by the Master of St. Katharine's, and carried unanimously, Lord Brassey proposed a resolution pledging the meeting to support the association to the utmost in the good work it was accomplishing, and, in doing so, said he desired most heartily to associate himself with the feeling of appreciation of and gratitude to Her Royal Highness for her characteristic graciousness in accepting the presidency of the association. He sincerely hoped that the public would make a generous response to the appeal for further subscriptions. The senior surgeon at the Middlesex Hospital, Mr. Henry Morris, seconded the resolution, and it was carried.

The Rev. Dacre Craven, in proposing a vote of thanks to the Duke of Westminster, said that for the past twenty-six years, ever since the association was inaugurated, indeed, the annual meeting had been held at Grosvenor House.

Lord Brassey proposed a vote of thanks to the Princess for presiding, and in doing so said Her Royal Highness could only be regarded as second to her late Majesty Queen Victoria, in the deep sympathy she felt for all movements which had for their object the alleviation of suffering. The vote was carried with applause.

### Everybody's Opinion.

**[Correspondence on all subjects is invited, but we cannot in any way be responsible for the opinions expressed by our correspondents. No communication can be entertained if the name and address of the correspondent are not given as a guarantee of good faith, but not necessarily for publication. All correspondents should write on one side of the paper only.]**

### WAITING ON THE KING'S GUESTS.

"Nurse M." writes: I was struck by a suggestion in Everybody's Opinion respecting the difficulty in getting people to wait on the King's poor guests. I would willingly give my services for the day should they have nurses to help.

"Nurse R." writes: I am a "Queen's Nurse" and belong to the R.N.P.F., and hope to be in London on my annual holiday at the time of the Coronation. I should be pleased to do as "Policy-holder 6855" suggests—help wait on the King's poor guests. Where should I apply?

"Policy 1079" writes: Having read in your paper that nurses are offering their services to wait on the King's guests, I write to say that I shall be in London during Coronation week, and I should be most pleased to give my assistance for the day should it be needed.

### THE TESTIMONIAL OF A MATRON.

"Discussion" writes: Is a matron of a general hospital at liberty to refuse a testimonial to a member of her staff who leaves at her own wish in order to take up work elsewhere, having during her service in the hospital given complete satisfaction to both matron and doctors, and receiving from the latter excellent testimonials? It seems to me most unfair, as there is always the chance of the matron retiring from work, or of her possible death to be considered. Supposing the sister or nurse so leaving does not intend entering at another hospital or institution directly, and a reference from either of these causes becomes impossible, the nurse or sister will inevitably suffer. As we all know, a matron's testimonial to a matron carries more weight than a doctor's.

### SLEEPING IN A PATIENT'S BEDROOM.

"R. J." writes: I should like to thank "Medicus" for his letter of April 26th. I have now been a private nurse for some years, but have been severely reprimanded because I would not go to cases where the male catheter had to be passed. I have never seen it done, and have always refused on that plea, but one matron actually suggested my learning on a dead patient. I only wish there were more doctors like "Medicus."

"M. H." writes: I have read the letter from "Medicus," in The Hospital of April 26th, with feelings akin to disgust. The first part of the letter deals with a question as to which there can be no hard and fast rule. He goes on to say, "my experience in private practice is limited"; had it been otherwise, that letter would never have been written. "If the medical man and the nurse were nice-minded enough?" needs but little comment. If they are not "nice-minded" they are unfit for any branch of their work. Personally, I think that "a nurse gets as much respect," and often more consideration, than other women. "False self-sacrifice" is said to be a woman's greatest fault, false modesty has surely no second place. Any nurse who is "squeamish" enough to refuse to do what lies in her power to alleviate human suffering, lacks not only modesty (in the true sense of the word) but sympathy. "Art" there may be none, but the comfort to the patient is of inestimable importance. Surely a "woman's feelings" will only be outraged when she suffers from the degrading taunts of those who think evil where there is none. To a nurse it is her work. "What do we live for, if not to make life less difficult for each other?"

"Fuzzy Wuzzy" writes: I can hardly say how pleased I was with the letter by "Medicus," *re* "Sleeping in a male patient's bedroom." He handled the matter so ably, particularly the "camel" part of the business. As to sleeping in a patient's room, it should not be. If a patient can dispense with the nurse's services so as to allow her to go to sleep, he does not want her at all, and therefore she should sleep in some other room; but let there be a bell by all means, or some other mode of communication, in case something unexpected happens. How any nurse can lie down and contentedly go to sleep in a patient's room, even with a screen intervening, I cannot understand. As to the other matter, *re* catheter, I quite agree with every word "Medicus" says, and as another correspondent remarks, "it is a growing evil." But now, when the opposite sex (and to our shame be it said) have taken the matter up and pointed out the, to say the least of it, indelicacy and unwomanliness of the proceeding, perhaps those women who boldly advertise for a male patient, and add "catheter" in their advertisement, may give up such a practice. But let me exhort all young nurses, or nurses just commencing their career, to absolutely refuse (if asked) to do anything of the kind, under a mistaken idea that it is part of a nurse's duty. I spent six years in a large general hospital, and no nurse in that hospital was ever expected to do such a thing, and even some other things which were taken for granted elsewhere. Consequently, all the nurses there were treated with respect both by doctors and patients, so they lost nothing by being a little "squeamish." I have been for nearly ten years a nurse, and I have never been asked to do such a thing except once, and that by a lay person and a "lady." I very soon showed her that there were nurses who had completed their training and who did not include passing male catheter in their qualifications. No medical man ever even suggested it to me, and I cannot help thinking that it must have been nurses themselves who first commenced, as "Medicus" rightly calls it, this "utterly degrading practice." Of course it is not to be expected that all doctors will be as good as "Medicus" in remembering a woman's outraged feelings of delicacy and modesty when she forgets them herself.

### THE CASE AGAINST HOSPITAL NURSES.

"X. Y. Z." writes: It goes sorely against the grain with me to admit that there can be anything seriously wrong with the profession of which I have been a member for many years, but it is as well to face facts, and there can be no doubt that private nurses are becoming increasingly unpopular. It has been asked, If so, why are they in so great request? I think that question can be answered by a remark made to me not long ago by a lady who had recently had a case of severe illness in her family. She said: "We could not have done without nurses, we realised and fully appreciated their skill; but, oh! I hope we shall never need another, they were so utterly wanting in tact and good manners. Quite recently I have heard of nurses asking for Burgundy in a house where money was obviously lacking; and, again, of two nurses who most rudely refused to take meals with the family because while their patient was in danger their meals were served in the study for lack of space in the dining-room, the sons and daughters having all come home. There is no use in multiplying instances, though one could do so almost indefinitely. Indeed, it is quite the exception to hear people speak happily of their trained nurses. It seems to be entirely a question of ethics, not of skill. I think, however, most people will agree with me that in Miss Johnston's charge against nurses, the word "Private" should have been used instead of "Hospital." As long as nurses are working in a hospital under the control of matrons and sisters, who are nearly always refined and educated gentlewomen, it is quite the exception to hear of rude and rough conduct; indeed it would not be tolerated; while to hear of hospital nurses being brutalised by overwork, tyranny, and bullying is, to those who know what hospital life really is in these days, almost comic. Will not private nurses lay these things to heart, and try to add to their undoubted skill the charm of courteous, refined, unselfish manners? I feel sure that if they were less self-assertive and less self-seeking, they would find that the friends of their patients would be easier to deal with, and they would not only have a better time generally, but they would also bring their profession into better repute.

### SHUTTING THE DOOR ON ROMAN CATHOLIC NURSES.

"Roman" writes: With regard to "Experience's" letter in a recent issue of your paper, I must say as a matron and a Roman Catholic, that I cannot agree with her when she states that Roman Catholic nurses require so much special leave for attendance at various services. As a rule, the number of them training at any institution is very small. Personally I have never found any difficulty in being able to arrange to attend church. By rising an hour earlier, it is easy to do one's share of the work, and then there is no inconvenience caused to anyone by going out to early Mass, which is not a long service. Undoubtedly, grave injustice is done to many Roman Catholics in some institutions. For instance, when I applied for a sister's post, after having been over three years in the hospital, the matron informed me that she should certainly object to having a Roman Catholic sister in charge, because of her influence on the patients and the nurses under her. Her bigotry completely blinded her to any other good points that I may have had as a nurse. Fortunately for me, the appointment did not rest with her entirely; the matter was taken out of her hands, and a nursing committee, consisting of members of the medical council and the general committee, selected me from a large number of applicants. One would have thought that a woman with her experience would have known that a sister in charge of a large ward has no time to preach religious doctrines, even if it so pleased her. I am sure that many others must have had similar experience to mine, and not with the same favourable result. When I applied for my present post, no question of religion arose. I have been told since by members of the committee that had it arisen objections would have been urged. I can now say with confidence, however, that in spite of my religion I have the fullest confidence of both the medical staff and the committee.

### Presentations.

East Malling District Nursing Association.—Miss Rhoda Loseby on resigning her post as district nurse at East Malling, near Maidstone, has been presented by her patients and friends with a handsome silver tea-set, pair silver backed brushes on bracket, a leather satchel, and many other presents.

### Wants and Workers.

Miss Moberly, Belsize Cottage, Belsize Lane, is despatching a parcel to No. 3 General Hospital, Kroonstad, O. R. Colony, on or about May 7th. She would be grateful for contributions of mittens, comforters, cardigans, or double yachting caps for the use of discharged patients.

### TRAVEL NOTES AND QUERIES.

Board in France (Nurse S.).—If you will send me a stamped and addressed envelope, I will give you the name of a cheap lodging where board can be had also.

Address in Paris (E. P.).—Thank you for the information you send me. I am always grateful for personal experience in the matter of hotels and lodgings.

### Echoes from the Outside World.

### Movements of Royalty.

THE King, the Prince and Princess of Wales, Princess Christian, Princess Louise, Duchess of Argyll, and Princess Henry of Battenberg visited Lord's Cricket Ground on Saturday, in order to witness the lacrosse match between the Canadian team and a strong side got together by the Duke of Argyll. A great many ladies were present. The progress of the game was watched with great interest by the Royal party, and the result was a decisive victory for the Canadians, whose score was 11 goals against 3 goals by the Duke of Argyll's team. As the King drove away from Lord's the Toronto men leaned out of the pavilion in order to cheer him as he passed. This, as the Duke of Argyll, presiding at a dinner the same evening in honour of the Canadian team, said, was the first time the King had visited Lord's since his accession.

Following the example set some years ago by his illustrious father, the Prince of Wales has decided that the gifts and addresses presented during his visit to the Colonies shall be exhibited to the public. The exhibition will take place at the Imperial Institute, and the proceeds will be contributed to the "Coronation Gift" to King Edward's Hospital Fund. It will be opened to the public on Thursday, May 15th, and will occupy the west half of the Special Exhibition Gallery of the Institute at South Kensington.

The Princess of Wales last week visited the annual exhibition of work organised by the "Society for Promoting Female Welfare by the United Working of Institutions for Women and Girls of Good Character," at the Albert Hall. Her Royal Highness inspected every stall, and purchased two sailor suits in white drill, with blue collars, from the Soldiers' Wives' Aid Society's stall; also examples of work done by the girls in the Princess Mary Village Home at Addlestone; articles from the stand of Mary Wardell Convalescent Home; and some woolly dolls exhibited by the Bristol and Clifton Association for the Blind.

### The Coronation.

THE Coronation Service is to be abbreviated by the omission of the "First Oblation," the Ten Commandments, the Hallelujah Anthem, and the final prayer. The Litany and the Benediction will be curtailed, and the peers' homage, which occupied a great deal of time at former coronations—each peer taking the oath and ascending the throne and touching the crown on the King's head and kissing his Majesty's cheek—is to be abbreviated by limitation of the personal act to the senior peer of each degree. The Coronation Oath itself is to be modified by the omission of all reference to the Church of Ireland, and there are alterations of certain anthems, designed to save time. Another omission from the ceremonial will be that of the throwing of gold and silver medals among the people in the Abbey, a form of largess bestowed by the King in former times through the medium of the Treasurer of the Household. In the matter of these medals the precedent of the Queen's Diamond Jubilee in 1897 will be followed.—The King and Queen will visit the City of London, in accordance with the custom observed by many of their predecessors, during the week following the Coronation, and will lunch with the Lord Mayor and Corporation at the Guildhall. Her late Majesty Queen Victoria visited the Guildhall on November 9th, 1837, after her accession. King George III. and his consort attended the 9th of November banquet in 1837, after his Majesty's accession, and the Court was celebrated with great magnificence. King George IV. declined the City's hospitality on his accession because reference was made in an address to the throne to the great increase of taxation which had been levied upon the industry of the people.

### Foreign.

THE latest bulletins respecting the condition of the Queen of Holland are favourable, and it is officially stated that the course of her Majesty's illness is in complete accord with the period which the malady has reached. So far, it is added, no complications have supervened. Unofficially, it is intimated that the Queen's condition is so far improved that she has been able to leave her bed for a short time.

The general election in France has resulted in a great success for the Ministry, and M. Waldeck-Rousseau has been afforded a new lease of power. This is, of course, contrary to the hopes of his opponents, but all Europe rejoices that a Prime Minister who has proved his capacity to govern so admirably as M. Waldeck-Rousseau should not only have retained his position, but received a clear vote of confidence which cannot fail to strengthen it in the eyes of France and of the world. M. Loubet has held entirely aloof from the election, in accordance with his conviction that the chief of the State ought to keep himself far above party politics. France has given a pledge to maintain the interests of peace.

### Music.

THE promised attendance of the Prince and Princess of Wales at the Queen's Hall on Friday last brought together a large concourse of hearers to listen to the musical performances of the pupils of the Royal Normal College for the Blind, and the concert was, in all respects, an excellent one. The choir sang "God Bless the Prince of Wales" as the royal patrons entered the room, and other vocal contributions included Mendelssohn's anthem, "Saviour of Sinners," Sir Hubert Parry's "Blest Pair of Sirens," and a couple of madrigals. One movement of Guilmant's First Symphony in D Minor for organ and orchestra was given, Miss Emily Lucas proving herself an organist of much talent. But perhaps the most interesting performance, because of his youth, was the rendering of the pianoforte part of the first movement of Beethoven's Concerto in E flat by Master Leonard Pegg. His correctness and execution were remarkable. The Princess graciously received purses on behalf of the institution, and some of the blind pupils themselves were amongst those who presented the offerings they had collected. The result was a substantial addition to the funds of the college.

### The Drama.

TWO interesting theatrical revivals took place on Saturday. Sir Henry Irving, having returned from his American tour, made his reappearance in "Faust" at the Lyceum, and received a most cordial welcome from a crowded house. His representation of Mephistopheles has lost none of the force which has always caused it to rank amongst his finest impersonations, and, like good wine, it has mellowed and improved with age. The scene in the garden when Mephistopheles, disguised as a nobleman, courts Martha—admirably played by Miss M. A. Victor—is especially noteworthy. Mr. Laurence Irving as Valentine gives a spirited performance, and Mr. H. B. Stanford as Faust looks the character to the life, has a fine voice, but is at present not sufficiently romantic for the part. The interest of the evening naturally centred around the Margaret of Miss Cecilia Loftus, who, it is stated, was selected and coached for the part by Miss Ellen Terry herself. Hitherto the name of Miss Loftus has been chiefly associated with clever mimicry, but she showed by her performance on Saturday that she has real histrionic gifts. She was far too nervous at times to do herself justice, but both her glee at the discovery of the jewel box, and her pathos in the prison at the end, were exceedingly well pourtrayed. The scenery is almost identical with that used in the original production nearly seventeen years ago, which has since been destroyed by fire.—"Caste," which was revived at the Haymarket Theatre on the afternoon of the same day, demonstrated forcibly the abiding popularity of Robertson's plays. The "Esther" of Miss Winifred Emery is as sweet and winsome as it could be made; the "Polly Eccles" of Miss Marie Tempest saucy and delightful; the "Sam Gerridge" of Mr. George Giddens racy and genial; and the "Eccles" of Mr. Cyril Maude admirable in its quiet humour.

### For Reading to the Sick.

THE CHRISTIAN'S GOOD NIGHT.

Sleep on, beloved, sleep and take thy rest,
Lay down thy head upon thy Saviour's breast;
We love thee well, but Jesus loves thee best;—
Good-night!

Only "Good-night!" beloved, not "Farewell!"
A little while, and all His saints shall dwell
In hallowed union, indivisible;—
Good night!

Until we meet before His throne
Clothed in the spotless robe He gives His own,
Until we know, even as we are known:—
Good-night!

*S. Doudney.*

For that day we all are, or ought to be, preparing. Some in the struggle of life here on earth, some in the silent calm of the world beyond the grave, we all have to look forward to the Great Day. Not in the Church Militant here on earth, not in the Paradise of waiting souls, is the perfection of the new creation of God. . . .

For that day our work in this life is meant to fit and prepare us. No true labour is in vain; no pure aim or noble purpose really fails; no trial or suffering need be fruitless; all are to have their outcome, their fruition, their satisfaction, in the perfected life, in the vision of God. "Wherefore, my beloved brethren, be ye steadfast, unmoveable, always abounding in the work of the Lord, forasmuch as ye know that your labour is not in vain in the Lord."

*J. W. Hicks.*

So we will plod on to the end; we will not ask to count God's gains. Hereafter, oh, how blessed the joy, if, indeed, by God's grace, it be given to any of us, to learn all the futility of our childish impatience, as we are shown by the Spirit the real harvest that Christ was ever reaping, off fields that once looked to us so desolate and barren. This may be ours hereafter if we be not all unfaithful to the blood of sprinkling; and for the present we will desire not to be taken out of the world, but to be kept from its evil. For the present let it be enough that the Lord direct our hearts unto the patient waiting for Christ.—*H. S. Holland.*

Yet there is blessedness that changeth not,
A rest with God, a life that cannot die,
A better portion, and a brighter lot,
A home with Christ, a heritage on high.

Hope for the hopeless, for the weary rest,
More gentle than the still repose of even!
Joy for the joyless, bliss for the unblest,
Homes for the desolate in yonder heaven.

The tempest makes returning calm more dear,
The darkest midnight makes the brightest star;
Even so to us, when all is ended here,
Shall be the past, remembered from afar.

*Rev. H. Bonar, D.D.*

Amen! until there shall be no more "days,"
Until the Shadows flee,
Until the cloud be lifted from our gaze,
Until in Certainty
Trust die, and Faith in Sight, and Prayer in Praise,
In God's Eternity!

*S. J. Stone.*

### Notes and Queries.

**The Editor is always willing to answer in this column, without any fee, all reasonable questions, as soon as possible.**
**But the following rules must be carefully observed:—**

1. **Every communication must be accompanied by the name and address of the writer.**
2. **The question must always bear upon nursing, directly or indirectly.**

**If an answer is required by letter a fee of half-a-crown must be enclosed with the note containing the inquiry, and we cannot undertake to forward letters addressed to correspondents making inquiries. It is therefore requested that our readers will not enclose either a stamp or a stamped envelope.**

*Horse-hair Mattresses.*

(42) My committee have asked me to get 50 horse-hair mattresses for the ward beds. Can you kindly tell me where would be the best place to buy them?—*Matron.*

We cannot recommend individual firms. There is nothing very special about horse-hair except to "see that you get it."

*Transverse Presentation.*

(43) In a case of transverse presentation is it possible for it to become head presentation without assistance?—*Bessie.*

We are told that in certain circumstances all things are possible, and no doubt in some infinitesimally small number of cases spontaneous rectification may take place. Such a chance, however, must never be counted upon. Treatment is urgently necessary.

*Morphia.*

(44) Can you tell me if it is possible to get a book giving information on morphia more than is given in the Pharmacopœia?—*Nurse Annie.*

Consult a book on materia medica, say that by Dr. Mitchell Bruce (Cassell and Co.).

*Engagement to Nurse.*

(45) I was engaged to nurse a lady for May 8th. The confinement has just come off, and being in the country the friends could not summon me in time. Can I claim anything?—*L. O. S.*

Half fees are usually paid in such cases; but as the friends in this instance did their utmost to give you the case, you can hardly claim them legally unless you had made a stipulation to that effect upon engagement. Maternity nurses should always do this.

*Rome.*

(46) I am anxious to have some details of the Anglo-American Nursing Home in Rome. I want to know if the nurses are employed in private nursing as well as in the home; and if the home is open all the year.—*H. L.*

Write, enclosing stamps, to the Matron, 256 via Nomentana, Rome.

*Floor Stain.*

(47) Can you recommend any cheap, light-coloured floor stain for the floors of a new house which is to be used as a hospital for one year only?—*Sister.*

A solution of permanganate of potass is as good as anything. It can be applied at any depth of colour preferred. The stain should be coated with oak varnish.

*Nurse for Boer Prisoners.*

(48) Can you tell me how to obtain information which will enable me to become a nurse at the Boer Refuge Camps?—*C. H.*

Apply to the Under Secretary, the Colonial Office, S.W.

*South Africa.*

(49) I have had two years' fever training in a London fever hospital. Will you kindly tell me if I should be able to get work in South Africa without general training, and, if so, where should I apply for it?—*District Nurse.*

There is no agency which will send you out to South Africa without a three years' certificate in general nursing.

**Standard Nursing Manuals.**

"The Nursing Profession: How and Where to Train." 2s. net; post free 2s. 4d.
"Art of Massage." (Second Edition.) 6s.
"Elementary Physiology for Nurses." 2s.
"Elementary Anatomy and Surgery for Nurses." 2s. 6d.
"Practical Handbook of Midwifery." 6s.
"Surgical Ward Work and Nursing." Revised Edition. 3s. 6d. net; post free 3s. 10d.
"Mental Nursing." 1s.
"Art of Feeding the Invalid." 1s. 6d.
All these are published by the Scientific Press, Ltd., and may be obtained through any bookseller or direct from the publisher, 28 and 29 Southampton Street, London, W.C.

## Figures and Tables

**Fig. 36. f1:**
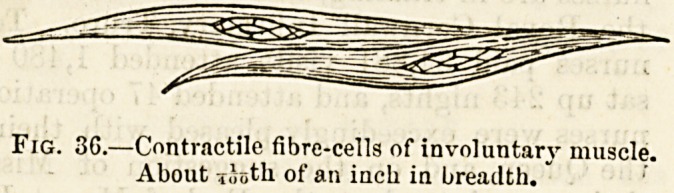


**Fig. 37. f2:**
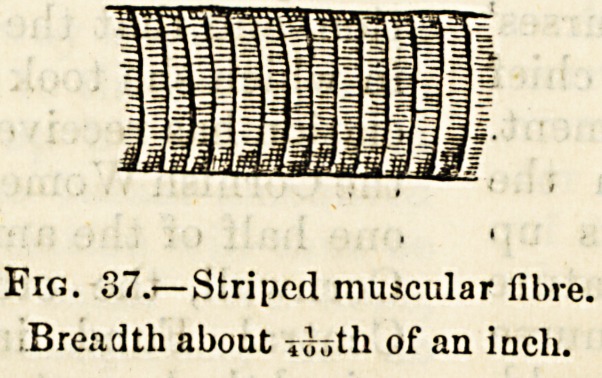


**Fig. 38. f3:**
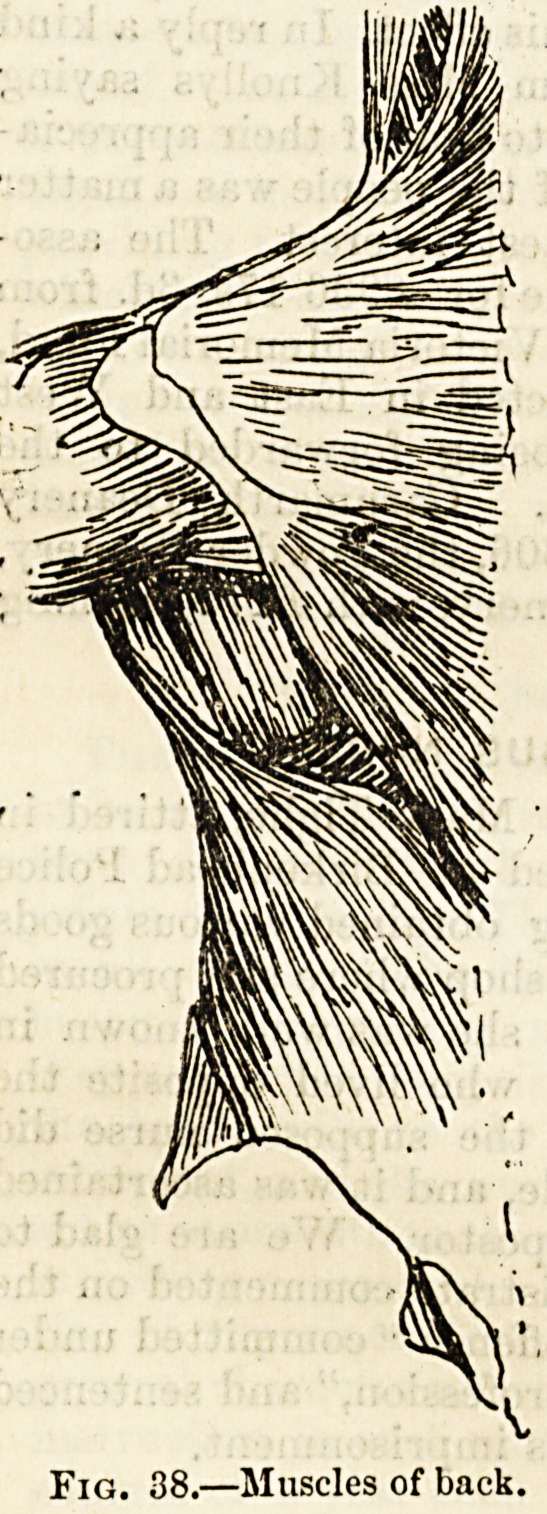


**Fig. 39. f4:**
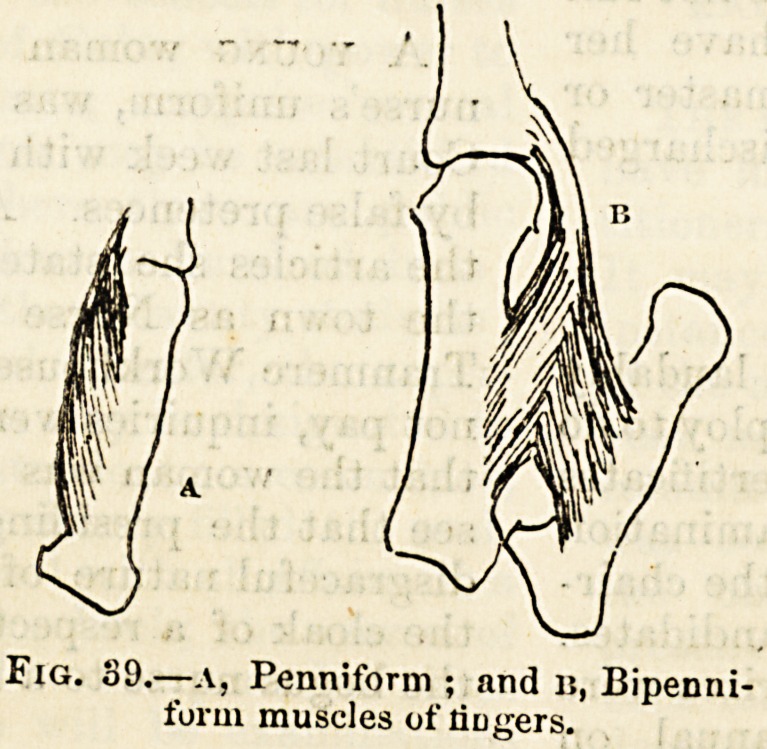


**Fig. 40. f5:**
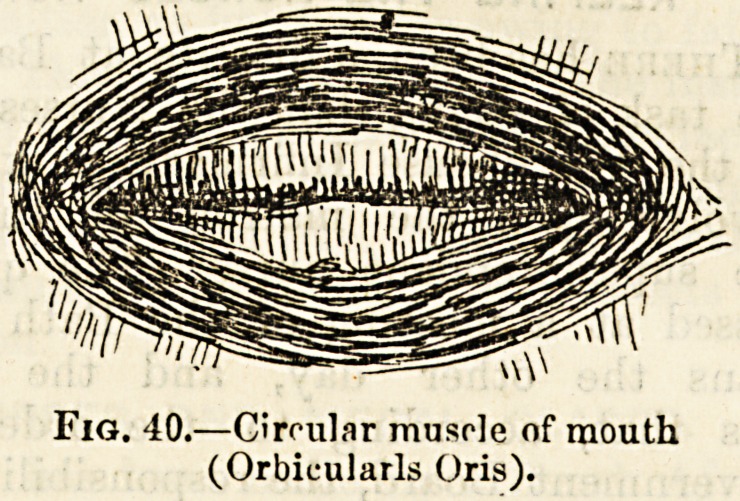


**Fig. 41. f6:**